# Lower Gastrointestinal Bleeding Associated With Variceal Hemorrhoidal Disease in a Patient With Pan-Colonic Varices

**DOI:** 10.7759/cureus.42013

**Published:** 2023-07-17

**Authors:** Gustavo Cervantes Millán, Rebeca Marina Tejeda Saenz, Maria E López Acosta, Javier Perez Aguirre

**Affiliations:** 1 General Surgery, Hospital Angeles Lomas, Huixquilucan, MEX; 2 Endoscopy/Gastroenterology, Hospital Angeles Lomas, Huixquilucan, MEX; 3 Coloproctology, Hospital Angeles Lomas, Huixquilucan, MEX

**Keywords:** lower gastrointestinal bleeding, case report, portal hypertension, idiopathic, hemorrhoidal disease, colonic variceal

## Abstract

Colonic varices are a rare condition primarily caused by portal hypertension associated with conditions such as cirrhosis or hepatocellular carcinoma. Idiopathic cases are even rarer, with less than 50 cases with a pan-colonic affection reported in the literature. Males are more commonly affected, with an average age of 41 years. Colonic varices can involve the entire colon in idiopathic cases and are often familial. Gastrointestinal bleeding is the main symptom, ranging from mild to life-threatening. Diagnosis is typically made through colonoscopy, which reveals dilated bluish vascular tracts. Treatment involves fluid IV resuscitation and controlling hemorrhage through various methods such as endoscopic procedures. Correction of the underlying cause is essential in cases of portal hypertension. Recurrent or unstable cases may require colon resection.

On this occasion, we present the case of a female patient who experienced profuse lower gastrointestinal bleeding. The patient's colonoscopy revealed the presence of varices throughout the entire length of the colon, with the only recent bleeding site being in the hemorrhoidal tissue. Therefore, a hemorrhoidectomy was performed to carry out an effective and less invasive therapeutic procedure than a colectomy with an excellent postoperative evolution.

## Introduction

The variceal disease of the digestive system is widely known and relatively common when it occurs with gastroesophageal involvement. However, colonic varices represent a rare condition that can cause various degrees of lower gastrointestinal bleeding [1]. In most cases, it is caused by portal hypertension, with a prevalence of 1%-8% when it is associated with a portal condition. However, when it is not associated with concomitant diseases, it is called idiopathic, with a prevalence rate of around 0.07% and less than 50 cases reported in the literature [1,2].

We present the case of a female patient with idiopathic pan-colonic varices and lower gastrointestinal bleeding due to variceal hemorrhoidal disease.

## Case presentation

A 56-year-old female patient presented to the emergency room with the antecedent of two episodes of lower gastrointestinal bleeding that required hospitalization years ago with no other relevant medical history. On this occasion, she began 12 days before her admission with glistening red rectorrhagia that was initially scanty and later became abundant. She also reported intense burning epigastric pain without irradiations, which subsided with the administration of proton pump inhibitors (PPIs), accompanied by nausea without vomiting. On the day of her admission, she had an episode of syncope and was taken to the emergency department.

Upon physical examination at admission, the patient was in stage II hemorrhagic hypovolemic shock, with a blood pressure of 70/30 mmHg and a heart rate of 93 bpm. Abdominal examination revealed a soft abdomen, tenderness on deep palpation in the epigastric region, and no signs of peritoneal irritation.

Initial blood tests showed a hemoglobin level of 10.7 mg/dL, hematocrit of 30%, mean corpuscular volume (MCV) of 86.6 fL, white blood cell count of 7.96 × 10^3^/µL, and platelet count of 249 × 10^3^/µL (Table 1).

**Table 1:** Initial blood tests. MCV: mean corpuscular volume, AST: aspartate aminotransferase, ALT: alanine transaminase, ALP: alkaline phosphatase, GGT: gamma-glutamyl transferase, LDH: lactate dehydrogenase, PT: prothrombin time, PT/INR: prothrombin time/international normalized ratio, aPTT: activated partial thromboplastin time, TT: thrombin time

Analyte	Patient's analyte
Leucocytes	7.96 × 10^3^/µL
Hemoglobin	10.71 g/dL
Hematocrit	30%
MCV	86.6 fL
Platelets	249 × 10^3^/µL
Glucose	89.5 mg/dL
Creatinine	0.73 mg/dL
Total bilirubin	0.23 mg/dL
Direct bilirubin	0.1 mg/dL
AST	33.9 U/L
ALT	48.2 U/L
ALP	58.5 U/L
GGT	22.6 U/L
LDH	145.6 U/L
Total protein	5.1 g/dL
Albumin	3.13 g/dL
Amylase	107 U/L
Lipase	72 U/L
PT	11.9 seconds
PT/INR	0.98
aPTT	26.5 seconds
TT	17.1 seconds
Sodium	136
Potassium	4.3
Chlorine	107

Due to the characteristics of the bleeding, the patient was initially diagnosed with upper gastrointestinal bleeding, with a Glasgow-Blatchford score of 6 points and a pre-endoscopic Rockall score of 2 points.

Treatment was initiated with fluid IV hydration with crystalloid solutions, normalizing vital signs, and empirical pharmacological treatment with PPIs. It was decided to admit her to the intermediate therapy unit for hemodynamic monitoring. Upper endoscopy was performed with findings that showed no evidence of a possible bleeding site.

**Figure 1:**
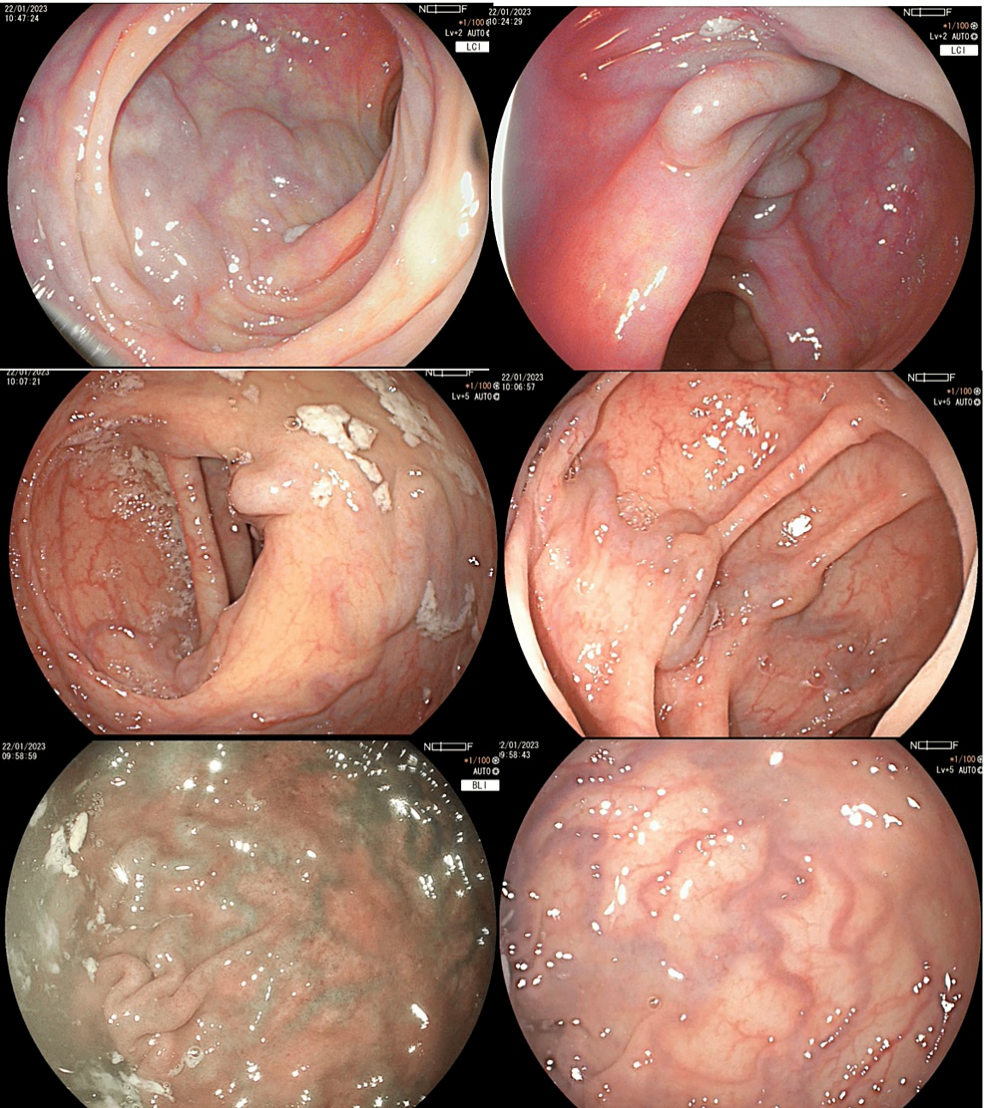
Pan-colonic variceal disease in our patient.

**Figure 2:**
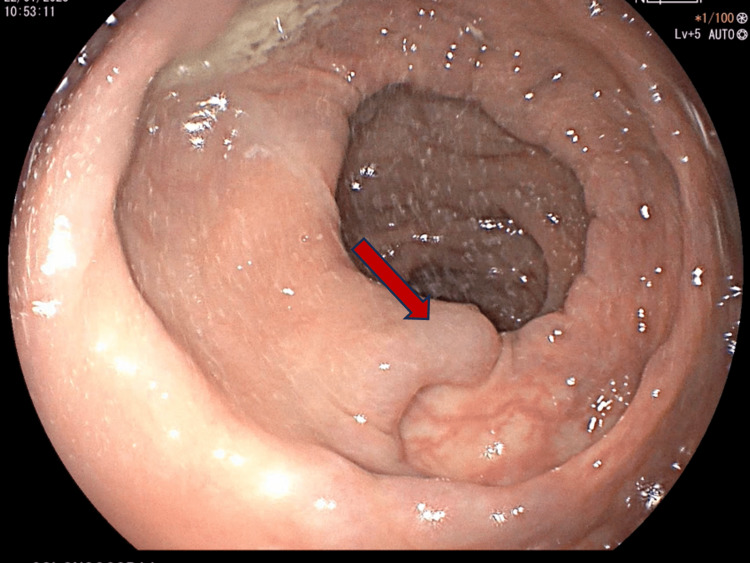
A 0.5 mm tubular adenoma on the descending colon (red arrow).

**Figure 3:**
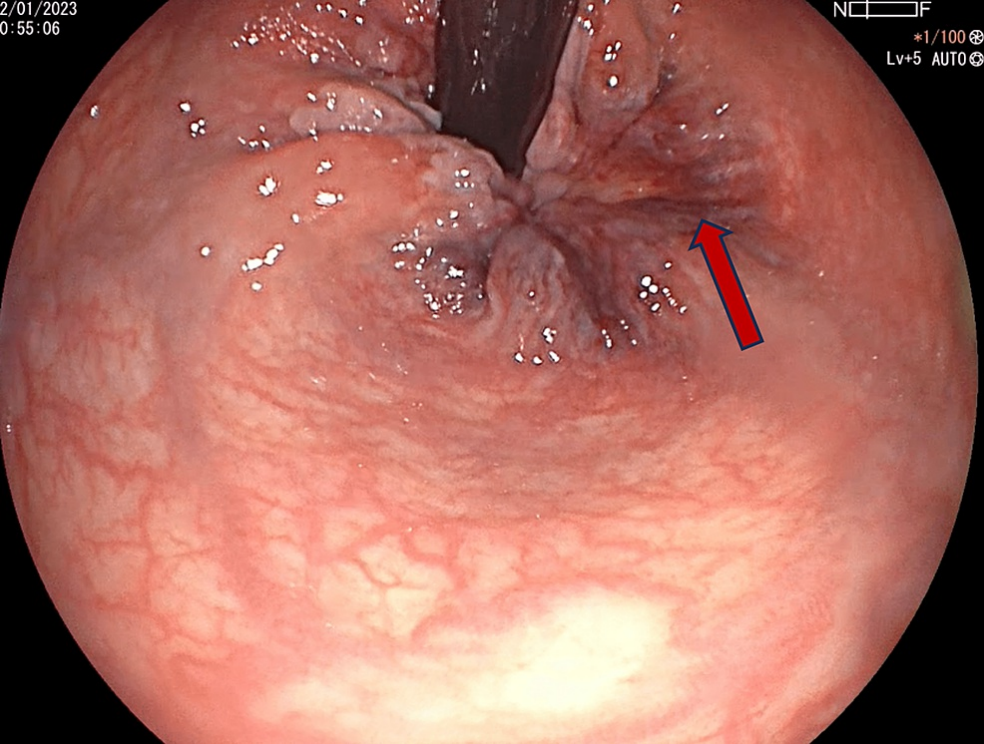
Variceal hemorrhoidal disease with evidence of recent bleeding and a mucosal laceration (red arrow).

The following day, hemoglobin levels decreased to 7.7 mg/dL with no data of active bleeding. A colonoscopy was performed in which dilated varices of the colon were observed at the ileocecal valve, cecum, ascendent, and transverse colon, with no data of recent bleeding (Figure 1) with regular shape, contractility, distensibility, haustral pattern, and 5 mm diameter adenoma with a short tubular pattern at the descending colon (Figure 2). A polypectomy was performed with biopsy forceps, and due to abundant bleeding, a hemoclip was placed. In the anorectum, a varicose hemorrhoidal disease was observed with a mucosal laceration and data of recent bleeding (Figure 3).

Lower gastrointestinal tract hemorrhage (Oakland score: 23 points) was diagnosed secondary to grade IV varicose hemorrhoidal disease.

**Figure 4:**
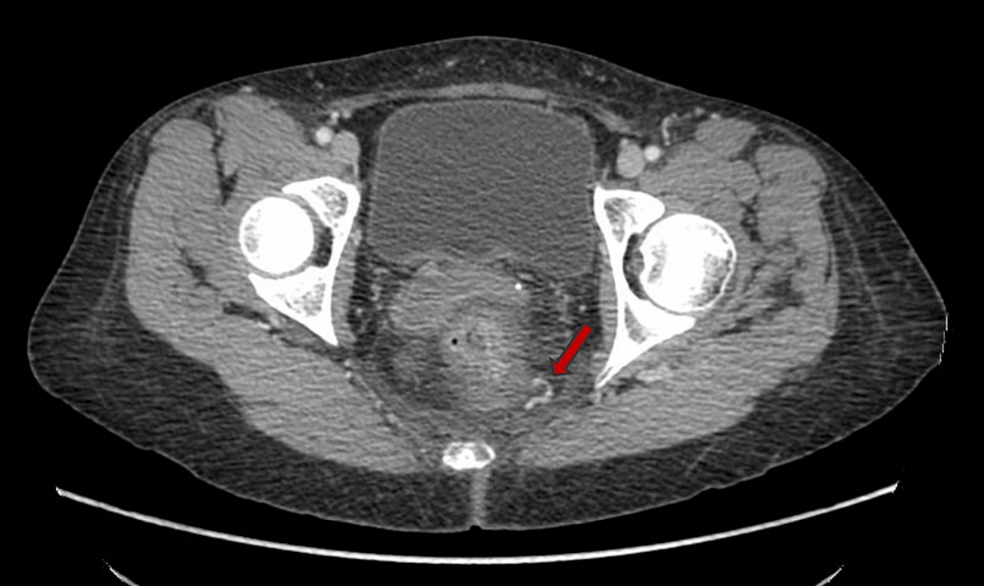
Dilated vein in the posterior wall of the rectum (red arrow).

**Figure 5:**
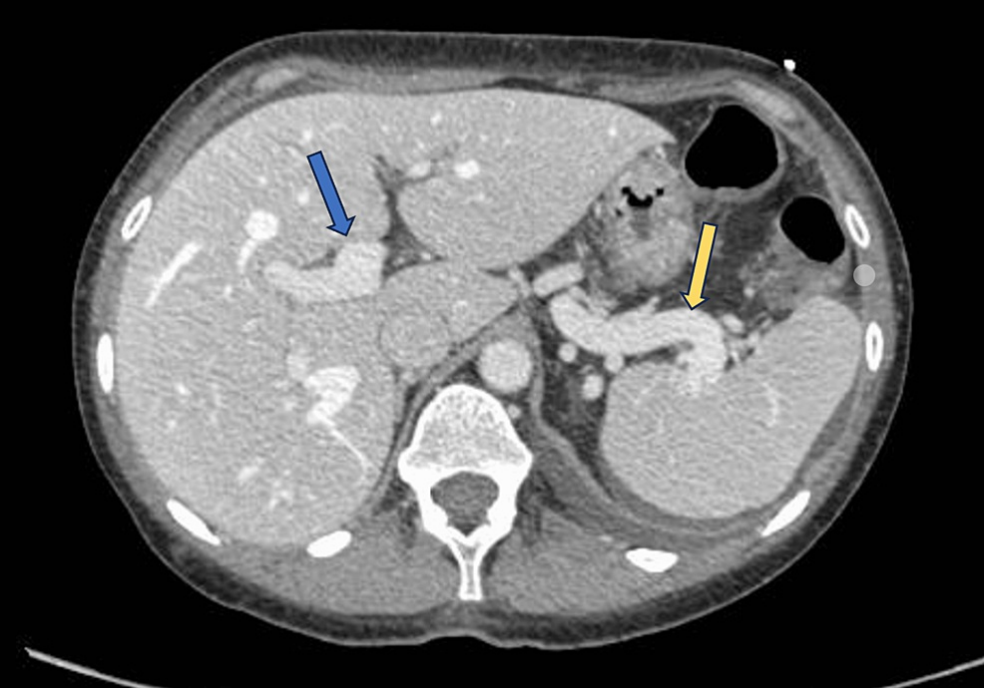
Tortuous and dilated portal (blue arrow) and epigastric (yellow arrow) veins.

An abdominal CT with IV contrast showed ectatic and tortuous veins toward the posterior wall of the rectum (Figure 4) and cecum, toward the region of the gastric antrum and distal ileum, as well as a significant increase in the caliber of the portal vein and splenic vein (Figure 5).

**Figure 6:**
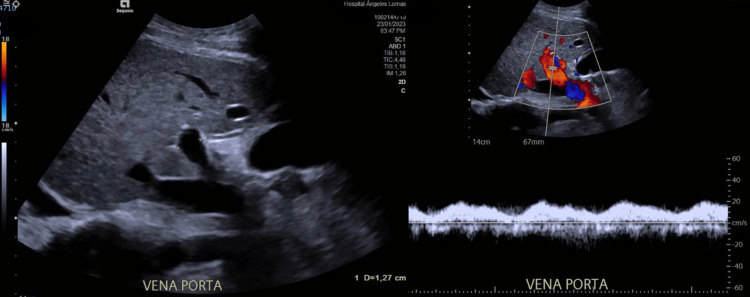
Dilated portal vein with normal flow.

Subsequently, an abdominal Doppler ultrasound was performed due to the suspicion of portal hypertension, which revealed the presence of portal vein dilatation with normal flow (Figure 6) and normal median acoustic radiation force impulse (ARFI) elastography (1.1 m/s) (Table 2).

**Table 2:** Hepatic elastography values (METAVIR). ARFI: acoustic radiation force impulse

METAVIR	ARFI (m/s)	kPa
No fibrosis F0	<1.3	-
Mild fibrosis F1	1.31-1.55	<7.1
Moderate fibrosis F2	1.56-1.7	7.1-8.8
Severe fibrosis F3	1.71-1.85	9.5-9.6
Cirrhosis F4	>1.86	12.5-14.6

Management with venotonics and iron infusion was started, and she continued with adequate clinical evolution until her discharge.

Subsequently, the patient underwent a hemorrhoidectomy in a second hospitalization to seek a definitive treatment without subjecting the patient to major surgery. The patient evolved adequately and has not presented a recurrence of bleeding four months after the surgical procedure.

## Discussion

Colonic varices are a rare condition with a general prevalence of around 0.07%, as reported by Feldman et al. in a study that autopsied 2,912 patients with gastrointestinal bleeding [3]. In most cases, they occur as a consequence of portal hypertension, primarily due to cirrhosis, hepatocellular carcinoma, or portal thrombosis [1-6]. However, fewer than 50 cases have been reported with an idiopathic presentation of pan-colonic involvement [1,2], with even lower prevalence rates. Males are more commonly affected, with a ratio of 25:15, and the average age of presentation is 41 years, ranging from 14 to 81 years [5,7].

In cases with non-idiopathic etiology, the involvement is generally segmental, affecting the right (cecum) or left (rectum) colon. However, in cases of idiopathic presentation, approximately 50% involve the entire colon. A familial form of the disease is diagnosed when multiple family members are affected [7]. In 2014, Speicher et al. published a study where out of 39 cases of colonic varices found in the literature, 26 had pan-colonic involvement, and 15 had a familial component [5]. In 2020, AlOmran et al. updated this case series with 49 reported cases, of which 37 had pan-colonic involvement, 12 had segmental involvement, and 18 had familial involvement [7].

Most patients with colonic varices are asymptomatic, but the main clinical feature is gastrointestinal bleeding, ranging from mild microscopic bleeding with chronic anemia to life-threatening massive hemorrhage [5,7-9].

While often diagnosed incidentally during studies for other causes, endoscopy is currently the diagnostic method of choice. It serves as the mainstay for diagnosing and treating patients with gastrointestinal bleeding in general. In cases of colonic variceal bleeding, a colonoscopy allows for the diagnosis by visualizing tortuous and dilated vascular tracts with a bluish color in the colon. It also helps determine the extent of the disease and perform treatment in most cases [6,7,9].

Other proper diagnostic methods for colonic varices include mesenteric angiotomography in the venous phase, with a sensitivity of up to 95% for detecting the bleeding source, and capsule endoscopy, which evaluates the entire digestive tract length [9].

Treatment, similar to other sites of gastrointestinal bleeding, involves resuscitation with intravascular volume replacement and maintaining hemoglobin levels above 8 g/dL. The following alternatives are available for controlling the bleeding: sclerotherapy, cyanoacrylate injection, and band ligation (endoscopic), simple suture ligation and portocaval shunt surgery (surgical), and embolization and endoscopic sclerotherapy assisted with a balloon (radiology intervention) [6,10].

In cases of variceal bleeding secondary to portal hypertension, the underlying cause should be corrected, and surgical treatment options may include a portocaval shunt. Patients with varices secondary to portal hypertension have higher mortality rates when undergoing subtotal or total colon resection compared to those undergoing portal decompression [7].

Nonoperative treatment should be considered with surveillance, iron supplements, laxatives, and blood transfusions in patients with single bleeding episodes without hemodynamic instability [7,11]. Definitive treatment should involve resection of the affected colon segment with partial or total colectomy in cases of recurrence or patients with hemodynamic instability [7,9,12]. The prognosis of idiopathic varices seems to be better at all ages than cirrhotic varices; this may be related to low pressure in the varices as the absence of chronic hepatic disease.

Patients with idiopathic colonic varices generally have a more favorable prognosis than those with varices secondary to portal hypertension [13].

Our patient presented with pan-colonic varices; however, the bleeding site was located at the anorectum with the context of hemorrhoidal variceal disease. A hemorrhoidectomy was performed to avoid major surgery, and the patient showed good postoperative recovery and no signs of another episode of hemorrhage six months after discharge but maintained close monitoring in case of a new episode of gastrointestinal bleeding that would require a total or subtotal colectomy.

## Conclusions

Colonic varices are a rare cause of gastrointestinal bleeding. Treatment should be individualized based on clinical characteristics and the patient's overall condition. However, it is accepted that surgical treatment can be considered to remove the affected colon area in cases of recurrent massive bleeding or hemodynamically unstable patients.
